# Rising congenital syphilis rates in Canada, 1993–2022

**DOI:** 10.3389/fpubh.2024.1522671

**Published:** 2025-01-17

**Authors:** Ashorkor Tetteh, Nadifa Abdi, Victoria Moore, Geneviève Gravel

**Affiliations:** Centre for Communicable Diseases and Infection Control, Public Health Agency of Canada, Ottawa, ON, Canada

**Keywords:** congenital syphilis, vertical transmission, prenatal care, maternal syphilis, prenatal syphilis, pregnancy, missed opportunities, Canada

## Abstract

**Introduction:**

The number of cases of confirmed early congenital syphilis has risen steeply in Canada in recent years, particularly since 2018, to the highest number ever recorded since national reporting began in 1993. We analyzed national data on confirmed early congenital syphilis from 1993 to 2022 to describe epidemiologic trends in Canada during this period.

**Methods:**

Data from 1993 to 2017 were obtained from routine surveillance conducted through the Canadian Notifiable Disease Surveillance System, and data from 2018 to 2022 were obtained from enhanced surveillance conducted through a federal-provincial-territorial working group. Case counts and rates were computed nationally and by province and territory. Infectious syphilis data from the same time period for females of reproductive age were also analyzed.

**Results:**

The national rate of confirmed early congenital syphilis was 127-fold higher in 2022 than in 1993, increasing from 0.3 to 32.7 cases per 100,000 live births. Case counts began increasing rapidly in 2018, with the highest case count observed to date (*n* = 115) occurring in 2022. The highest rates in the country in recent years have been observed in Saskatchewan, Manitoba, Alberta, and Ontario. Infectious syphilis rates among females of reproductive age have also been rapidly increasing in these provinces. Between 2018 and 2022, the national rate of confirmed early congenital syphilis increased approximately seven-fold and the national rate of infectious syphilis increased approximately two-fold, including an approximately three-and-a-half-fold rate increase among females of reproductive age.

**Discussion:**

These numbers represent huge shifts in the epidemiological landscape of syphilis in Canada. The increase in vertical transmission appears to be driven by not only the increasing rate of infectious syphilis among females of reproductive age but also by multiple structural and social determinants of health impacting pregnant individuals.

## Introduction

Canada has experienced a major surge in cases of congenital syphilis in recent years, reporting the highest numbers observed since national reporting began in 1993 (1).

This surge is concurrent with rising infectious syphilis rates that have had a disproportionate impact on gay, bisexual, and other men who have sex with men (GBMSM) in the past several decades and are now also heavily impacting heterosexual populations and, in particular, females of reproductive age (2). The rising rates of syphilis in the general population are not happening in a vacuum but rather occurring together with rising rates of other sexually transmitted and blood-borne infections (STBBIs) such as chlamydia, gonorrhea, hepatitis B, and hepatitis C. (3–6).

Infectious syphilis rates in Canada have risen steeply since 2018, impacting the number of infected pregnant individuals (i.e., cases of prenatal syphilis) passing the bacteria on to their babies *in utero* through vertical transmission, resulting in congenital syphilis (1).

The surge in cases of congenital syphilis is also observed in other high-income countries that have had historically low case counts. The United States reported 3,761 cases of early congenital syphilis (both confirmed and probable) in 2022, 10 times more than in 2012 (7, 8), when cases started to increase (9). It also reported an additional 231 syphilitic stillbirths in 2022. Australia, which first reported an outbreak in 2011 in the state of Queensland (10), reported 12 cases of congenital syphilis (confirmed and probable live births and stillbirths) in 2022, six times more than in 2016 (11, 12). New Zealand first started to experience an increase in cases of congenital syphilis in 2017 (13) and reported six cases of early congenital syphilis (unclear whether confirmed and/or probable) in 2022, six times more than in 2016. It also reported two additional syphilitic perinatal deaths (including one stillbirth) (14, 15). England, which first started to experience an increase in cases of congenital syphilis in 2016 (16), reported six cases of confirmed early congenital syphilis in 2022, a fifth more cases than in 2016 (17). Finally, Japan, which has been experiencing an increase in congenital syphilis cases since 2014 (18, 19), reported 20 cases of confirmed early congenital syphilis in 2022, a third more cases than in 2016 (20, 21).

A fetal syphilis infection can have grave consequences, including miscarriage or spontaneous abortion, premature birth, and stillbirth, as well as significant and multisystem involvement manifesting with a myriad of clinical symptoms in newborns. Early congenital syphilis can have its first manifestations up to the age of 2 years after birth while late manifestations can appear in childhood. In all cases, long-term sequelae are common (22–24).

Yet, congenital syphilis remains a preventable disease that can be avoided with timely testing and diagnosis followed by adequate treatment during pregnancy (24, 25). To meet the need for timely testing and diagnosis, the Public Health Agency of Canada (PHAC), the leading governmental authority for public health surveillance in Canada, has published clinical guidelines, developed in consultation with the National Advisory Committee on STBBI (NAC-STBBI), that as of 2022 recommend universal screening in the first trimester or at the first prenatal visit and, in areas with outbreaks or for pregnant people at ongoing risk of infection or reinfection, repeated screening at 28–32 weeks of pregnancy and again at delivery. It is also recommended that individuals at high risk and those with a history of a perinatal loss by stillbirth (≥ 20 weeks of gestation) undergo more frequent screenings (26). Given the current epidemiology, the NAC-STBBI guidelines are currently under review to evaluate universal repeated screening. In provinces and territories (PTs), guidelines currently vary in their repeated screening recommendations. Congenital syphilis is also preventable with adequate treatment, which NAC-STBBI guidelines define as having received 2.4 million units of benzathine penicillin G LA injected intramuscularly according to a dosage schedule appropriate for the stage of syphilis infection and completed at least 4 weeks before delivery, with a sufficient reduction in the pregnant individual’s non-treponemal titres and no evidence of reinfection (27, 28).

In 2022 and earlier, only confirmed cases of early congenital syphilis were nationally reportable to PHAC, limiting this study to an analysis of congenital cases of *Treponema pallidum* infection that were diagnosed within the first 2 years of life and that have been confirmed in a laboratory, according to the national case definition in use at the time (Supplementary Table S1) (29). The data on confirmed early congenital syphilis analyzed in this study thus represent an underestimation of the true disease burden in Canada, because cases that are not captured by national, provincial, or territorial reporting are not included in this study. These include cases for which data collection is sparse or data are unavailable due to the absence of certain jurisdictional case classifications, poor data quality among the data collected, or failure to consider a congenital syphilis diagnosis as a result of cases being asymptomatic or hard to ascertain and thus not prompting health care providers to order laboratory testing. For these reasons, national or jurisdictional reporting may exclude a variety of cases, including probable cases, syphilitic stillbirths, syphilis-induced miscarriages, asymptomatic cases of congenital syphilis; and late-diagnosed (≥ 2 years of age) cases of congenital syphilis; or cases that may be missing a documented link as a syphilis-related outcome, as may occur with premature births and neonatal and infant deaths due to congenital syphilis (3, 25, 30, 31).

The diagnosis of congenital syphilis is inherently complex, more so than the diagnosis of syphilis in adults, for several reasons. First, signs and symptoms of congenital syphilis may be difficult to clinically detect following birth as they may not yet have developed, and when they do, they may not be specific enough (32). Most (60–90%) newborns are asymptomatic and may only present with signs and symptoms during the first few months of life (by 3 months of age) or even after 1 year of age (33, 34). Secondly, congenital syphilis may also be difficult to confirm by laboratory testing either because the infection is recent and not yet detectable, as in the case of birthing parents who acquire the infection late in their pregnancy, or because the trans-placental passage of antibodies from the parent to the child complicates the interpretation of serologic tests for up to 18 months following birth (32). In this context, a false negative test result may occur in an infant with congenital syphilis when their infection is recent and testing has occurred within the window period of antibody development (30). As such, the majority of cases of congenital syphilis are diagnosed based on the birthing parent’s medical history, comparisons between newborn and birthing parent serologic titres, and clinical judgment even in the absence of laboratory confirmation of infection in the newborn. In clinical practice, given the severe consequences of not treating suspected cases of congenital syphilis, and the long period of follow-up required to obtain a confirmatory diagnosis, infants may be treated pre-emptively (7, 32, 35). Fortunately, the use of sensitive direct testing methods such as nucleic acid amplification tests (NAATs) makes it possible to obtain a definitive diagnosis, however its cost can be prohibitive (36). Thirdly, human immunodeficiency virus (HIV) co-infection in a pregnant individual, causing impairment of cell-mediated immunity, may lead to syphilis serologies being falsely negative or positive, seroconversion being delayed, or treatment response being inadequate, all of which have the potential to negatively impact congenital syphilis diagnostic timelines, test accuracy and pathological outcomes in infants (31, 33). Of note, HIV is not the only infection that can cause false positive or false negative test results – other bacterial, viral, spirochetal, plasmodial, rickettsial, and protozoal infections or diseases can also cause false positives, though at varying degrees of frequency in the general population (37–39). Furthermore, HIV co-infection in pregnant individuals increases the risk of transplacental syphilis transmission as well as the risk of transplacental HIV transmission, indicating the need to consider both HIV and syphilis testing in the event of suspicion of either disease (33, 40).

Ultimately, successfully connecting pregnant individuals to prenatal care, timely testing and diagnosis, and adequate treatment requires an elucidation of the drivers for the increasing incidence of infectious syphilis among these individuals, so as to better understand and address missed opportunities for intervention in their prenatal care. This approach will enable public health actors to more effectively combat the rising cases of congenital syphilis in Canada.

The aim of this study is to describe epidemiologic trends in Canada for confirmed early congenital syphilis since it became nationally notifiable in 1993.

## Methods

### Data sources

Confirmed cases of sexually acquired or, rarely, blood-transfusion acquired syphilis and confirmed cases of congenital syphilis have been notifiable in Canada since 1924 and 1993, respectively (1). For this study, data from 1993 to 2017 on the infectious stages of syphilis (primary, secondary, early latent, and infectious neurosyphilis) and on congenital syphilis were extracted from the Canadian Notifiable Disease Surveillance System (CNDSS). Data from 2018 to 2022 were extracted from PT data submissions to the Syphilis Outbreak Investigation Coordinating Committee (SOICC), a federal-provincial-territorial (FPT) working group whose mandate includes national enhanced surveillance of syphilis. The variables submitted by PTs to CNDSS and SOICC and used in the analyses were as follows: infectious syphilis case count, congenital syphilis case count, age, sex, year, and PT. The national case definition for congenital syphilis in use up to 2022 is provided in Supplementary Table S1 (29). It has since been revised and can be found online (28).

### Data analyses

Case counts and crude rates were computed both nationally and by PT, stratified by year of diagnosis, age group at time of diagnosis, and/or sex, as relevant. PTs with case counts less than 10 in a given year were excluded from PT-stratified analyses (but included for Canada-wide analyses) for that year to protect the privacy of individuals. Females of reproductive age are defined in the analyses, in conformity with CNDSS age group categories, as females 15 to 39 years old, which differs from most PTs’ and the World Health Organization (WHO)‘s definition (i.e., females 15–49 years old) but captures 95% of births in Canada (1, 41). Infectious syphilis rates were calculated per 100,000 population (either total population, males, females, or females 15–39 years old), and congenital syphilis rates were calculated per 100,000 live births, using as numerators reported case counts and as denominators yearly estimates based on the latest Statistics Canada population estimates released in June 2024 and the latest Statistics Canada live birth estimates released in September 2023 (42, 43). Enhanced surveillance data for one PT in 2020 and for two PTs in 2021 were projected, due to incomplete data submissions, by doubling the case counts for the first two quarters to estimate case counts for the full calendar year. The proportion of missing data from reporting PTs for sex and age did not exceed 0.2% on average between 1993 and 2022. All descriptive analyses were conducted using Microsoft Excel (44).

Quantitative analyses were also conducted to describe the relationship between infectious syphilis among females 15–39 years old and congenital syphilis. Spearman rank correlation tests were performed on national and PT data, supported by statistical inference to test the null hypothesis of zero association. Furthermore, for national data only, due to the adequate sample size, Poisson regression models were fitted to the congenital syphilis case counts, with female infectious syphilis cases as the explanatory variable and the logarithm of live births as an offset term; and negative binomial regression models were used to generalize the Poisson regression for data exhibiting overdispersion. Finally, for both national and PT data, cross-correlation plots were used to assess the possibility of a lagged correlation or, in other words, whether a previous year’s increase/decrease in infectious syphilis would better predict congenital syphilis case counts in a given year. The analyses were conducted for six PTs only, due to too-few congenital syphilis case counts in the remaining PTs. Missing values for 2 years of data from Nova Scotia were imputed as zero cases in the analysis. No adjustments were made for underreporting or by age group, and no other explanatory variables were included in the analyses. The analytical tests selected are appropriate for the existing monotonic trends during the study period (1993–2022) and may not be suitable for other types of trends. All quantitative analyses were conducted using R version 4.3.2 (45).

## Results

### National epidemiology of confirmed early congenital syphilis and infectious syphilis among females of reproductive age

**Figure 1:**
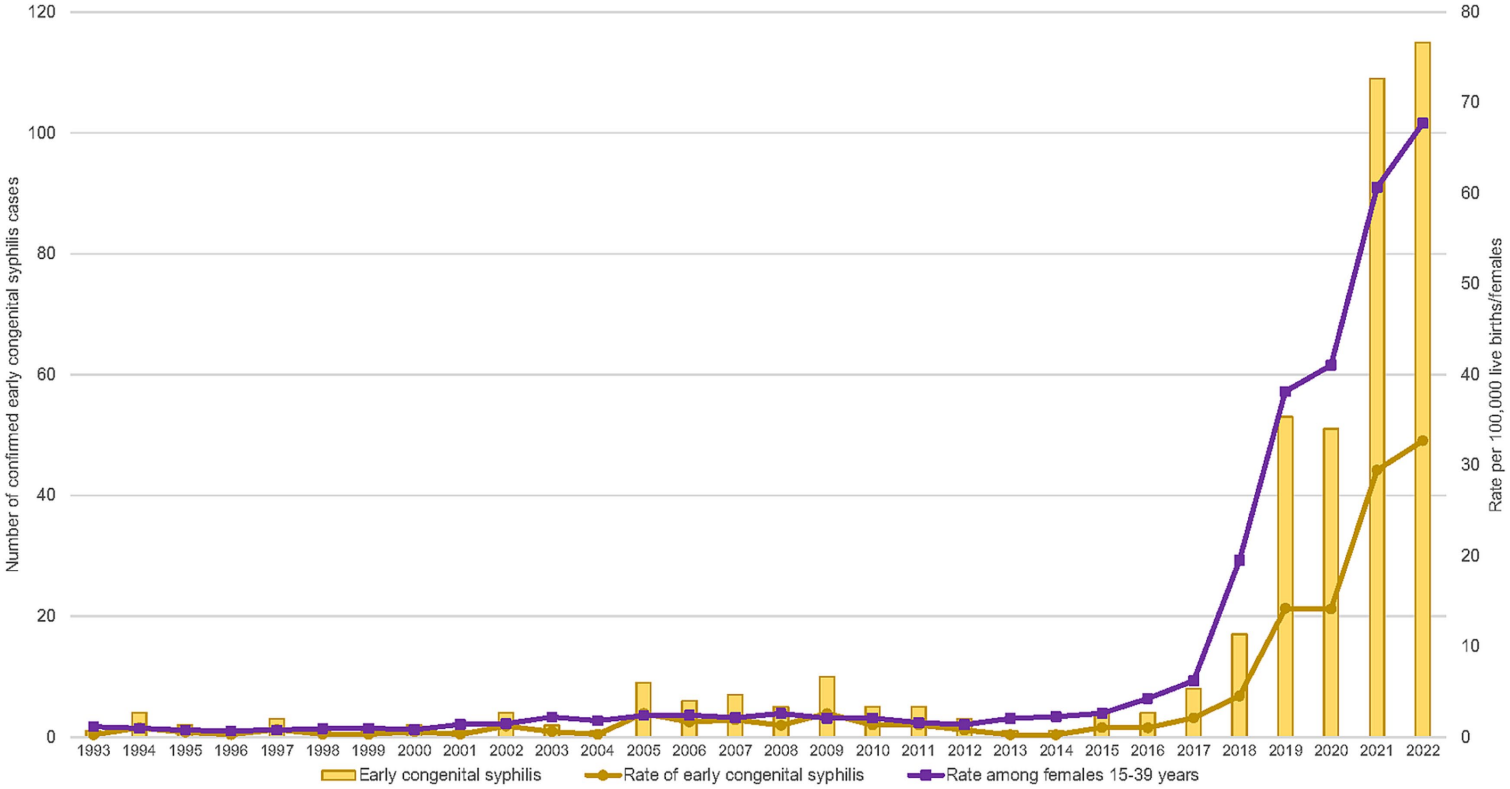
Number and rates of reported cases of confirmed early congenital syphilis and rates of reported cases of infectious syphilis among females 15–39 years old in Canada, 1993–2022.

The number of yearly cases of confirmed early congenital syphilis reported nationally was relatively low between 1993 and 2004, ranging from one to four cases and corresponding to a rate of 0.3 to 1.2 cases per 100,000 live births (Figure 1 and Supplementary Table S2). Then, in 2005, nine cases of confirmed early congenital syphilis were reported, corresponding to an approximately eight-and-a-half-fold rate increase (2.3 rate difference) compared to 2004, and this was followed by a fluctuation of reported case counts in subsequent years. In 2009, there was a record number of 10 cases reported (2.6 cases per 100,000 live births), followed by a decrease in case counts for the next 8 years. However, since 2018 when there were 17 cases of confirmed early congenital syphilis, cases have been rapidly increasing, with the highest-ever case count and rate of 115 cases and 32.7 cases per 100,000 live births, respectively, reported in 2022. The 2022 rate is 127-fold higher than the 1993 rate, representing an increase from 0.3 to 32.7 cases per 100,00 live births. The 2022 rate also represents an approximately seven-fold rate increase (28.2 rate difference) since 2018.

The rate of infectious syphilis among females 15 to 39 years old follows a similar trend to what was observed with congenital syphilis and also corresponds with the increase in cases of infectious syphilis in the population as a whole. Infectious syphilis cases rose to 14,135 cases in 2022, with a rate of 36.5 cases per 100,000 population. This represents an approximately two-fold rate increase (19.3 rate difference) since 2018. Among females 15 to 39 years old, specifically, relatively low rates were observed between 1993 and 2002, ranging from 0.7 to 1.5 cases per 100,000 females 15 to 39 years old (Figure 1 and Supplementary Table S2). Then, in 2003, the rate increased to 2.2 cases per 100,000 females 15 to 39 years old but remained relatively stable for over a decade (between 1.4 and 2.6 cases per 100,000 females 15 to 39 years old). However, the rate of infectious syphilis among females 15 to 39 years old has been increasing rapidly since 2016, with the highest jump in rate from the previous year observed in 2018 (an approximately three-fold rate increase; 13.3 rate difference) and the highest-ever rate observed in 2022 (67.8 cases per 100,000 females 15 to 39 years old). The 2022 rate represents an approximately three-and-a-half-fold rate increase (48.3 rate difference) relative to 2018.

### Jurisdictional epidemiology of confirmed early congenital syphilis

**Figure 2:**
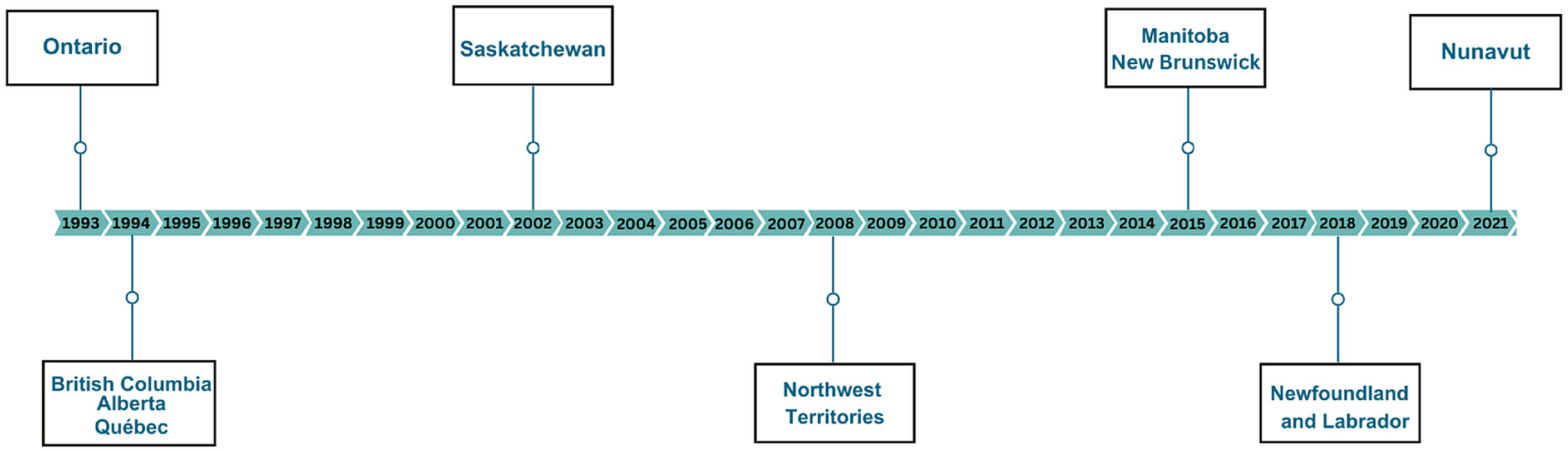
Timeline of reporting of the first case of confirmed early congenital syphilis in Canada.

In terms of jurisdictional trends, the first confirmed case of early congenital syphilis since congenital syphilis became notifiable in Canada in 1993 was reported in Ontario in 1993, followed by British Columbia, Alberta, and Quebec in 1994 (Figure 2). Subsequently, Saskatchewan reported their first case in 2002, followed by Northwest Territories in 2008. There was a 7-year gap before any other PTs (Manitoba and New Brunswick) reported their first confirmed case of early congenital syphilis. More recently, Newfoundland and Labrador and Nunavut reported their first cases in 2018 and 2021, respectively. Up to 2022, Yukon, Prince Edward Island, and Nova Scotia had not reported any confirmed cases of early congenital syphilis.

**Figure 3:**
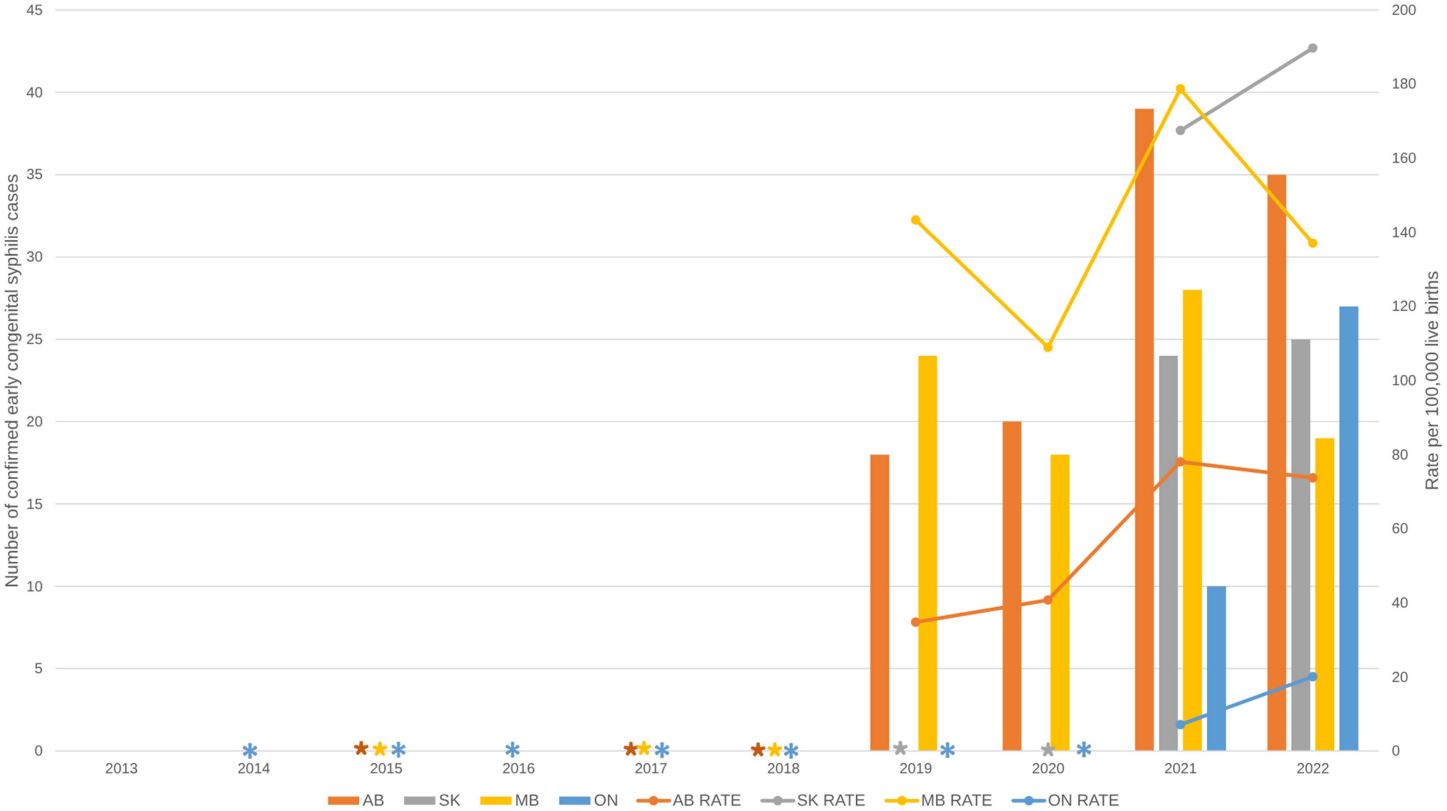
Reported number of cases and rate of confirmed early congenital syphilis by province and territory, 2013–2022. Data are based on the four provinces that have reported 10 or more cases of confirmed early congenital syphilis in Canada: AB, SK, MB, ON. All other provinces and territories reported fewer than 10 cases of confirmed early congenital syphilis and were excluded from the figure to reduce the risk of identifying individuals or are represented with asterisks if they reported 10 or more cases in later years. AB, Alberta; SK, Saskatchewan; MB, Manitoba; ON, Ontario.

From 1993 to 2018, all PTs reported less than 10 yearly cases of confirmed early congenital syphilis (Figure 3). However, since 2019, Alberta and Manitoba have been reporting more than 10 cases of confirmed early congenital syphilis, with the highest and second-highest number of cases ever reported in a single PT occurring in Alberta (39 cases) and Manitoba (28 cases), respectively, in 2021. Saskatchewan and Ontario have reported more than 10 cases since 2021, with each reporting their highest-ever number of cases in 2022 (25 and 27 cases, respectively). Overall, Alberta reported the highest case count of confirmed early congenital syphilis in the country between 2020 and 2022 (Figure 3).

In terms of rates, the highest rates in recent years occurred in Manitoba, Saskatchewan, and Alberta (Figure 3). In Manitoba, rates of congenital syphilis fluctuated but remained high between 2019 to 2022, with the highest rate (178.7 cases per 100,000 live births) occurring in 2021. Meanwhile, Alberta consistently had the second-highest rate in the country between 2019 and 2020, but was surpassed by Saskatchewan in 2021. In 2022, the rate in Saskatchewan (189.7 cases per 100,000 live births) surpassed the rate in Manitoba (137.1 cases per 100,000 live births) and became the highest rate reported to date among PTs with 10 or more cases of congenital syphilis. While rates in Ontario are lower relative to rates in the Prairie provinces of Manitoba, Saskatchewan, and Alberta, they increased sharply between 2021 (7.1 cases per 100,000 live births) and 2022 (20.0 cases per 100,000 live births) (Figure 3).

### Jurisdictional epidemiology of infectious syphilis among females of reproductive age

**Figure 4:**
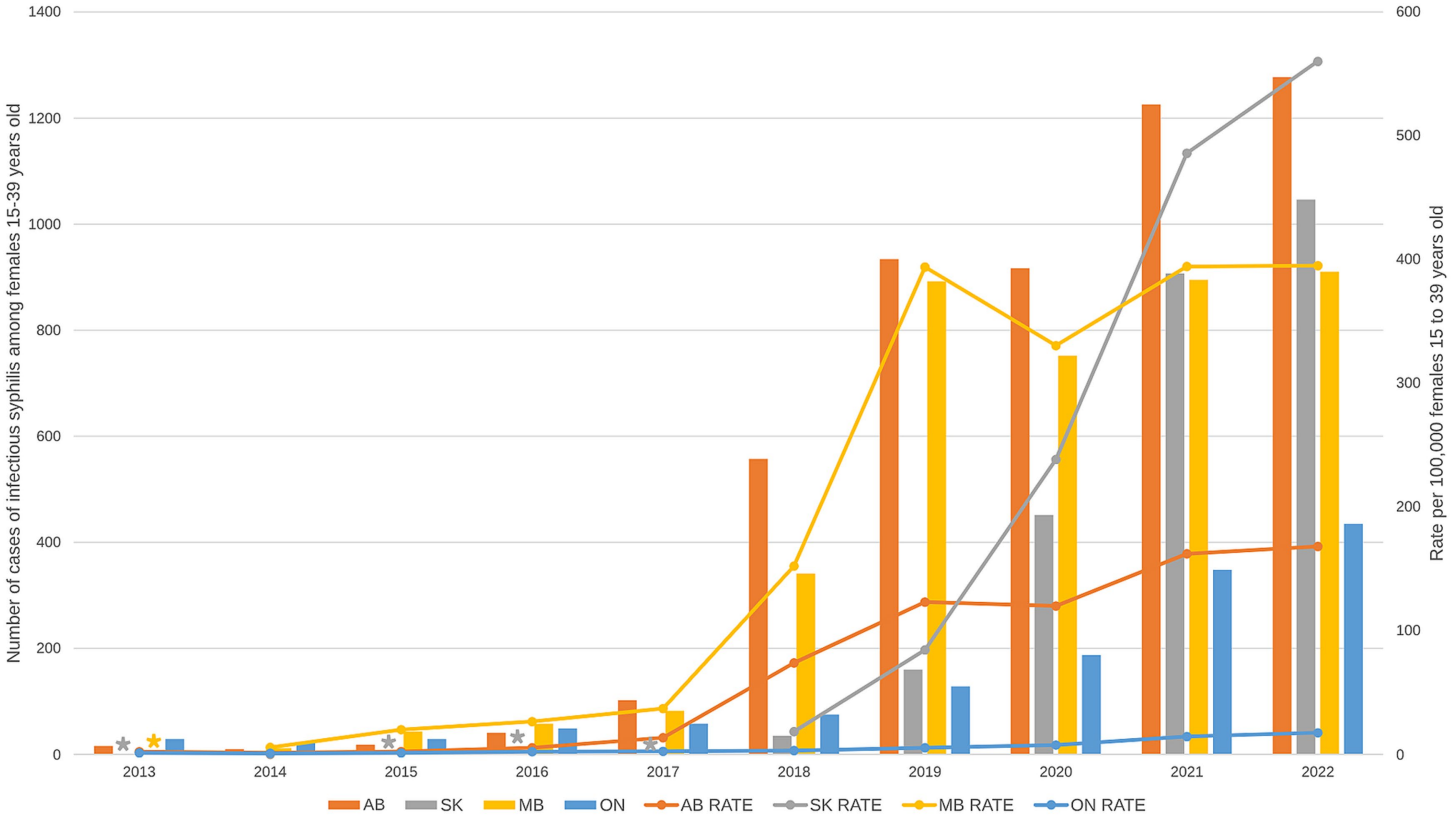
Reported number of cases and rate of infectious syphilis among females 15 to 39 years old by province and territory, 2013–2022. Data on infectious syphilis are shown only for the four provinces that have reported 10 or more cases of confirmed early congenital syphilis in Canada: AB, SK, MB, ON. Provinces and territories reporting fewer than 10 cases of infectious syphilis were excluded from the figure to reduce the risk of identifying individuals or are represented with asterisks if they reported 10 or more cases in later years. AB, Alberta; SK, Saskatchewan; MB, Manitoba; ON, Ontario.

In PTs that reported 10 or more cases of confirmed early congenital syphilis, case counts and rates have been increasing among female populations in the past decade, with a faster increase observed among those 15–39 years old, as has historically been the trend (2). Alberta and Ontario have reported 10 or more cases of infectious syphilis among females 15–39 years old since 2013, Manitoba has reported 10 or more cases since 2014, and Saskatchewan has reported 10 or more cases since 2018. Overall, rates among females 15 to 39 years old were highest in Manitoba until 2020, after which they were surpassed by rates in Saskatchewan in 2021. Alberta accounted for the second-highest rate until 2019, after which it was surpassed by Saskatchewan in 2020. Between 2020 and 2022, Alberta accounted for the third-highest rate but continued to account for the highest case count in the country of infectious syphilis among females 15 to 39 years old. The three Prairie provinces have been experiencing a steep increase in their rates of infectious syphilis among females 15 to 39 years old since 2018. While Ontario’s rates of infectious syphilis among females 15 to 39 years are considerably lower than rates in the Prairies, its cases continue to rise, with case counts nearly two times higher in 2021 (*n* = 348) than in 2020 (*n* = 187) and its highest case count reported in 2022 (*n* = 435) (Figure 4).

### Age group-specific trends among females of reproductive age

**Figure 5:**
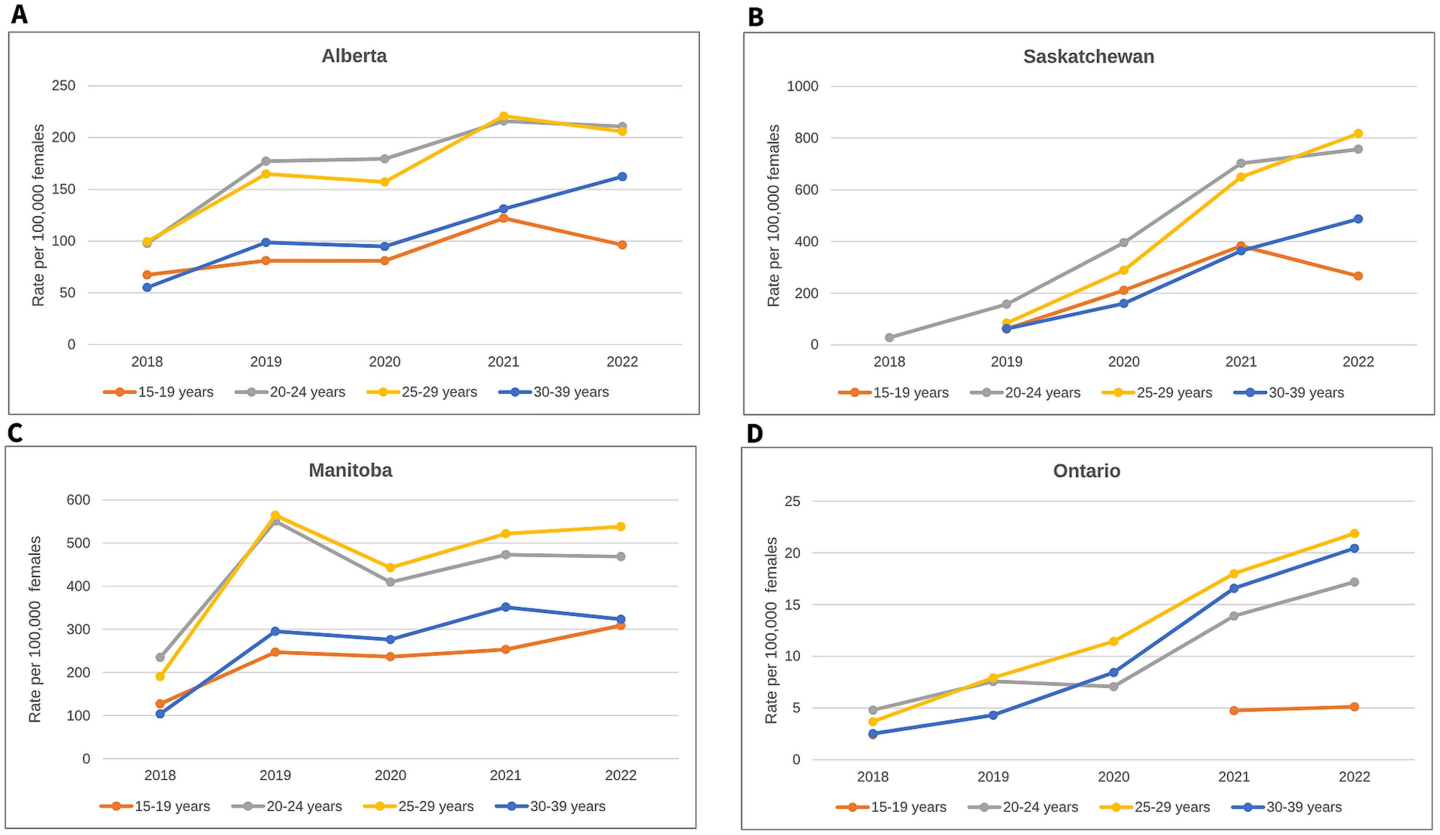
Rates of infectious syphilis among females 15–39 years old by age group for select provinces, 2018–2022: **(A)** Rates in Alberta; **(B)** Rates in Saskatchewan; **(C)** Rates in Manitoba; **(D)** Rates in Ontario. Data on infectious syphilis are shown only for the four provinces that have reported 10 or more cases of confirmed early congenital syphilis in Canada. Caution should be used when comparing rates across provinces as different scales are used for each graph.

While rates of infectious syphilis are rapidly increasing among females 15 to 39 years old, the magnitude of this increase has been more pronounced in particular age groups in the last few years (Figure 5 and Supplementary Table S3). Rates have been considerably higher among females 20 to 24 and 25 to 29 years old in Alberta, Saskatchewan, and Manitoba (Figures 5A–C) Alberta and Manitoba observed this shift in 2018, with rates nearly twice as high in the 20-to-24-year age group (97.7 and 234.5 cases per 100,000 females 20 to 24 years old, respectively) and the 25-to-29-year age group (99.4 and 190.2 cases per 100,000 females 25 to 29 years old, respectively) compared to those in the 30-to-39-year age group (55.2 and 103.7 cases per 100,000 females 30 to 39 years old, respectively). In Saskatchewan, a similar shift was initially observed in 2019 in females 20 to 24 years old, closely followed in 2020 by females 25 to 29 years old. Rates in Ontario were highest among those 20 to 24 and 25 to 29 for a number of years; however, since 2020, rates among the 30-to-39-year age group have surpassed rates in the 20-to-24-year age group (Figure 5D). Between 2018 and 2022, the highest rates among females 15 to 39 years old in each PT consistently occurred among those 25 to 29 years old, with peaks at different time points (Figure 5): in 2021 in Alberta (220.9 cases per 100,000 females 25 to 29 years old); in 2022 in Saskatchewan (817.5 cases per 100,000 females 25 to 29 years old); in 2019 in Manitoba (564.6 cases per 100,000 females 25 to 29 years old), and in 2022 in Ontario (21.9 cases per 100,000 females 25 to 29 years old) (Figure 5).

### A comparison of trends in infectious syphilis among females of reproductive age to trends in confirmed early congenital syphilis

**Figure 6:**
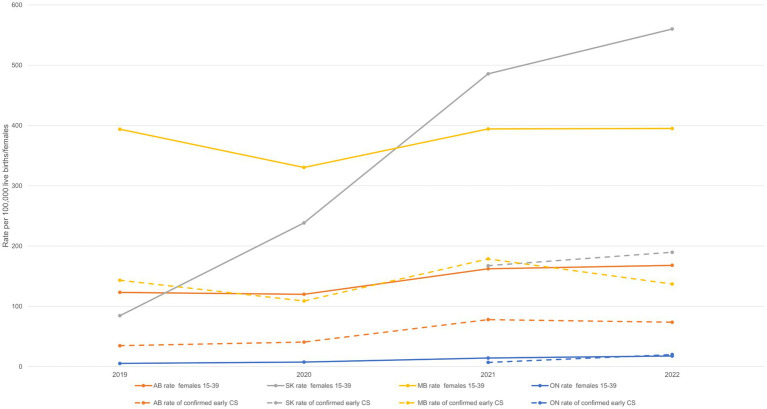
Rates of infectious syphilis among females 15–39 years old and rates of confirmed early congenital syphilis by province and territory, 2019–2022. Data are based on the four provinces that have reported 10 or more cases of confirmed early congenital syphilis in Canada: AB, SK, MB, ON. Provinces and territories reporting fewer than 10 cases of confirmed early congenital syphilis were excluded from the figure to reduce the risk of identifying individuals. AB, Alberta; SK, Saskatchewan; MB, Manitoba; ON, Ontario; CS, congenital syphilis.

As previously mentioned, Manitoba had the highest rate of confirmed early congenital syphilis in Canada from 2019 to 2020, after which it was surpassed by Saskatchewan, and Alberta also went from having the second- to the third-highest rate in 2021 (Figure 6 and Table 1). This trend is nearly identical to the trend observed among females 15 to 39 years old, whereby Manitoba had the highest rate of infectious syphilis in this female age group between 2019 and 2020, after which it was surpassed by Saskatchewan in 2021, and Alberta went from having the second- to the third-highest rate in 2020 (Figure 6 and Table 1). Thus, the trend in rates of confirmed early congenital syphilis has closely mimicked the trend in rates of infectious syphilis among females of reproductive age, as PTs with the highest rates of infectious syphilis among females 15 to 39 years old have also experienced the highest case counts and rates of congenital syphilis and, interestingly, are also characterized by the lowest male-to-female rate ratios of infectious syphilis (indicating the highest proportion of females affected, relative to males) in the country (Figure 6 and Supplementary Table S4).

**Table 1:** Rate of infectious syphilis among females 15–39 years old and rates of confirmed early congenital syphilis by province and territory, 2019–2022.

Year	Rate of infectious syphilis per 100,000 females 15 to 39 years old				Rate of confirmed early congenital syphilis per 100,000 live births			
	AB	SK	MB	ON	AB	SK	MB	ON
2019	123.2	84.5	393.9	5.4	34.8	DNS	143.3	DNS
2020	120.0	238.4	330.0	7.7	40.8	DNS	109.0	DNS
2021	162.2	485.8	394.3	14.4	78.1	167.5	178.7	7.1
2022	168.1	560.1	395.1	17.6	73.7	189.7	137.1	20.0

Data are based on the four provinces that have reported 10 or more cases of confirmed early congenital syphilis in Canada: AB, SK, MB, ON. Provinces and territories reporting fewer than 10 cases of confirmed early congenital syphilis were excluded from the figure to reduce the risk of identifying individuals. DNS: data not shown to reduce the risk of identifying individuals (low case counts). AB, Alberta; SK, Saskatchewan; MB, Manitoba; ON, Ontario.

The question of infectious syphilis rates in females 15–39 years old driving confirmed early congenital syphilis rates was explored through quantitative analyses of trends for both, between 1993 and 2022. The results confirmed a positive correlation between the two, with changes in infectious syphilis case counts among females of reproductive age being synchronous (no time lag within a given year) with changes in confirmed early congenital syphilis case counts over time. Nationally, the association was strongly positive ( $\hat{\beta }$ = 0.531, *se* = 0.01779, *p* < 0.0001), and in the regions, the association was weaker for Ontario (r = 0.531, *p* = 0.0025) and strongest for the three Prairie provinces (r = 0.580–0.797, *p* = 0.0008-< 0.0001).

## Discussion

The rise in the number of cases of confirmed early congenital syphilis has occurred in tandem with the rise in cases of infectious syphilis among females 15–39 years old, with a sustained high jump in case counts from 2018 onward (Figure 1). The Prairie provinces of Alberta, Saskatchewan, and Manitoba, in addition to Ontario, are bearing the brunt of high caseloads for confirmed early congenital syphilis, while the territory of Yukon and the Atlantic provinces of Nova Scotia and Prince Edward Island are notable in their absence of cases of confirmed early congenital syphilis up to 2022 (Figures 2–4). In 2022, Alberta had the highest case counts and Saskatchewan had the highest per population rates of both confirmed early congenital syphilis and infectious syphilis among females 15 to 39 years old (Figures 3, 4). Among females of reproductive age in the hardest-hit PTs, it is the younger end of the age spectrum, namely 20-to-29-year-olds who are the most impacted by syphilis transmission, although in Ontario, as of 2020, it is females 25 to 39 years old who have the highest rates (Figure 5).

Canada has committed to reducing these case counts by aligning with the WHO’s 2030 global targets for lowering rates of infectious and congenital syphilis. These targets aim for 80% of countries to have 200 or fewer cases of confirmed and probable congenital syphilis live births and stillbirths per 100,000 live births per year by 2025, and 50 or fewer cases of confirmed and probable congenital syphilis live births and stillbirths per 100,000 live births per year by 2030 (46). With rates rising to as high as 32.7 cases of confirmed early congenital syphilis per 100,000 live births in 2022 (Supplementary Table S2), Canada still has much work to do to reduce rates and advance overall health and wellbeing while minimizing disparities, in order to meet the 2030 global targets (47).

It is important to note that rates from 2020 to 2022 should be interpreted with caution as they occurred in the context of the coronavirus disease (COVID-19) pandemic (48), which included a period of decreased demand for and access to STBBI services, including prevention, testing, treatment, and other supportive services (49). This likely contributed to fewer detected and reported cases of infectious and congenital syphilis between 2020 and 2022. Conversely, some increases in case counts during this period may be influenced by increased health care demand following the reopening of STBBI services after lockdowns or may reflect higher disease transmission as a result of individuals with reduced access to care having a longer opportunity to transmit the infection to their sexual partners and/or changing the frequency of new sex partners or entering new sexual networks (50). As different parts of the country experienced various levels of disruptions due to COVID-19 from 2020 to 2022, comparisons of reported infectious and congenital syphilis case counts and rates between the PTs during this period may also be inaccurate.

National surveillance within a federated governance model has limitations with respect to monitoring and parsing the relevant risk factors driving epidemiological trends. Therefore, a systematic literature review focusing on Canadian studies was conducted to investigate the structural and social determinants of health as well as clinical and individual risk factors influencing current trends in prenatal and congenital syphilis rates.

Based on the literature review, the most impactful factors influencing current trends in prenatal and congenital syphilis rates were revealed to be a lack of timely and repeated prenatal syphilis screening (51–53), a lack of adequate treatment and follow-up of syphilis infection during pregnancy (53–59), barriers to accessing health care, including prenatal care, caused by multiple intersecting social determinants of health as well as by certain structural determinants of health (1, 2, 56, 60–62), and substance use (53, 55–57, 60, 63–65).

Many of these factors explaining the current trends in congenital syphilis are modifiable, some more easily than others, particularly when it comes to addressing missed opportunities for intervention in the patient journey of a pregnant individual. From a public health perspective, the results from the literature review highlight opportunities to improve the prevention of congenital syphilis in Canada, including through the revision of clinical screening guidelines for pregnant individuals and through improvements to public health surveillance.

### Opportunities to improve clinical screening guidelines

PT prenatal screening guidelines, along with NAC-STBBI recommendations, play an important role in supporting congenital syphilis prevention efforts. Recently, several PTs updated their prenatal screening guidelines to include universal screening during the third trimester and/or at delivery in addition to universal screening during the first trimester or at the first prenatal visit (66).

A precaution to note is that although additional screening can help prevent congenital syphilis (66), some research indicates that enhanced screening can also result in an elevated detection of cases and, consequently, an increase in observed rates (67). Since updating their prenatal screening guidelines for syphilis in 2019, British Columbia and Manitoba have experienced a rapid increase in the number of confirmed cases of early congenital syphilis in their jurisdictions (68, 69). A study evaluating the impact of British Columbia’s updated guidelines reported an increase in both prenatal and congenital syphilis cases following the implementation of the new recommendation (64). Another study examining the impact of Manitoba’s updated guidelines also suggested that intensified screening could uncover additional cases of congenital syphilis (67). The study noted a marked shift in testing patterns, where the proportion of pregnant individuals undergoing only one test dropped from 93% in 2015 to 57% in 2019, and the proportion of pregnant individuals receiving two or more tests rose from 7% in 2015 to 43% in 2019 (67). This highlights the importance of recognizing regional differences in screening practices to better understand the factors driving rapid rate increases for congenital syphilis. In the long term, however, earlier and more frequent screening of pregnant individuals, despite the potential for higher detection rates of prenatal syphilis, should lead to a lower incidence of congenital syphilis, indicating effective prevention of vertical transmission.

### Opportunities to improve public health surveillance

There is also an opportunity to improve efforts to prevent congenital syphilis through the strengthening of surveillance systems. National public health surveillance enables the monitoring of disease incidence with the aim of assessing epidemiological patterns and risk factors to support the development of prevention and control strategies and programs. As cases of congenital syphilis increased in Canada, it became evident that the existing case classification for confirmed early congenital syphilis was insufficient to adequately capture the burden of disease associated with congenital syphilis. An FPT task group was thus convened in 2022 to review and address issues identified in the existing case definition for congenital syphilis. Task group members reflected a range of expertise including laboratory medicine, microbiology, epidemiology, and clinical infectious disease. Members considered congenital syphilis and syphilitic stillbirth case classifications used in Canadian PTs as well as internationally and consulted current guidance documents and relevant scientific literature for discussions.

As of January 2024, the existing congenital syphilis case definition has been updated. The sole confirmed early congenital syphilis case classification it comprised has been modified by adding further criteria to confirm cases. These are: (1) infant’s rapid plasma reagin (RPR) titre at least four-fold higher than the mother/birthing parent’s RPR titre in samples collected during the immediate postnatal period, and (2) persistent positive treponemal serology in a child older than 18 months of age. Furthermore, four new congenital syphilis case classifications have been added, namely confirmed late congenital syphilis, probable early congenital syphilis, confirmed syphilitic stillbirth, and probable syphilitic stillbirth. The revised national congenital syphilis case definition can be found online (28).

These proposed changes to the case definition aim to increase its sensitivity and thus more fully capture the incidence of congenital syphilis in Canada, particularly for probable cases that cannot be confirmed due to missing information, stillbirths linked to syphilis during pregnancy, and late diagnoses. The revised national case definition further intends to provide an opportunity for PT alignment through the voluntary uptake and integration of the new case definition of congenital syphilis into routine processes by jurisdictions. This will improve comparability between jurisdictions and the understanding of congenital syphilis from a national perspective.

Beyond the use of case definitions, it is necessary to monitor additional variables in public health surveillance. These variables may pertain to individual determinants of health such as personal, social, economic, and environmental factors, or to health outcomes. Monitoring clinical factors such as the timing of engagement in prenatal care, syphilis screening, and completion of and response to treatment for syphilis would enable a better understanding of where issues are occurring in obtaining timely testing and treatment. Furthermore, the following factors must be considered: patient values and preferences, feasibility and acceptability of care, equity, and potential harms.

Enhancing regional surveillance can support public health authorities in making evidence-based policy and program decisions (70). As such, there are ongoing efforts to enhance regional surveillance in several jurisdictions across Canada, including revising case report forms to better monitor epidemiological trends and risk factors.

Unfortunately, enhanced surveillance data do not enable in a reliable way the documentation of some key information related to access to care, such as factors that are associated with fears of stigma, discrimination, or legal consequences when accessing health care, for some pregnant individuals. Surveillance data must thus be supplemented with research studies (including qualitative research), literature reviews, and conversations with stakeholders and experts (medical, community-based, or having lived experience). Such information equips public health policy and programming actors to adapt their responses accordingly.

### Strengths and limitations of national surveillance

National enhanced surveillance of congenital syphilis has several benefits. It allows for the monitoring of epidemiological trends across all PTs to inform program and policy responses. Effectively, surveillance generates the evidence that supports the development of prevention and control strategies and programs at the federal government level, enables interjurisdictional comparisons of situational contexts, informs the allocation of funds to public health actors across jurisdictions, and informs the development of national clinical guidelines, among other uses. It also makes it possible to obtain data in a timelier manner than through routine surveillance and to visualize projected trends based only on partial-year data. In addition, enhanced surveillance data collection is relatively flexible and can easily accommodate additional variables, although the ability to collect these is dependent on variations in availability, format, and data processing capacity across jurisdictions.

The national enhanced surveillance system however has several limitations as well. First, limiting data collection to only confirmed cases of early congenital syphilis creates an incomplete picture of the total burden associated with congenital syphilis since, as discussed previously, numerous cases go unreported. Other limitations include incomplete data on certain variables and the fact that, in general, data on some variables are not collected systematically and consistently across jurisdictions. Furthermore, surveillance and research on social determinants of health, including race, ethnicity, and Indigenous identity, would be helpful in better understanding the epidemiology of congenital syphilis within the diversity of the Canadian population. The COVID-19 pandemic, for instance, highlighted the value of disaggregated data, including disaggregation by race and ethnicity, in better describing epidemics, appropriately targeting interventions, and more efficiently allocating resources (71, 72). However, data collection on ethno-cultural identity is still challenging in Canada for various reasons, including data governance concerns (73). Finally, more integration of the national surveillance data systems would greatly enhance data-reporting efficiencies as well as public health responses and decision-making capabilities at both federal and PT levels.

### Study limitations

The results of the data analyses presented earlier should be interpreted with caution for several reasons. First, due to periodic updates of surveillance data by jurisdictions, historical counts and rates may change over time. Second, rate trends for confirmed early congenital syphilis and for infectious syphilis among females 15 to 39 years old may be subject to fluctuation, because when population denominators are small, even small changes in case counts can result in large rate changes. Furthermore, the exclusion of PTs with case counts below 10 from the analyses for privacy reasons decreases the robustness of statistical inferences by possibly missing trends in these populations. Lastly, as already mentioned, national surveillance is able to monitor very few risk factors, which makes it necessary to look beyond surveillance to research studies to better monitor risk factors associated with epidemiological trends. The epidemiological changes described therefore cannot be fully explained by the surveillance data alone, due to the lack of systematic analysis of individual health behaviors or other risk factors and their impact on trends.

## Conclusion

The rapid increase in cases of congenital syphilis and infectious syphilis, particularly among females 15–39 years old, represents a huge leap in the epidemiological landscape of syphilis in Canada. The approximately seven-fold increase in the rate of congenital syphilis since 2018 is impacted by the three-and-a-half-fold rate increase in infectious syphilis among females of reproductive age, and more specifically by factors impacting pregnant individuals such as barriers to screening, treatment, follow up, and health care access, as well as multiple intersecting structural and social determinants of health and substance use. To supplement these findings, we suggest conducting future longitudinal research studies to attempt to further quantify, through multiple regression models or path analysis, the relative contribution of these factors to the rise in cases of congenital syphilis. It would also be a good complement to have studies that attempt to quantify the potential impact of COVID-19 on case counts.

PHAC remains committed to working collaboratively with partners and stakeholders across the country to address infectious and congenital syphilis as a growing public health concern. Moving forward, PHAC will continue to prioritize coordinated actions to reduce syphilis rates among pregnant individuals as well as among non-pregnant adults and adolescents, while continuing to address the ongoing need for targeted interventions among GBMSM. This includes executing a coordinated FPT plan for action on syphilis, continuing to improve national enhanced surveillance of congenital syphilis through the implementation of new case classifications and the collection of prenatal care variables, jointly supporting syphilis-related research initiatives with the Canadian Institutes of Health Research, reviewing screening guidelines in collaboration with PTs, and leveraging community partnerships to provide grants and contributions funding to community-based organizations that are addressing syphilis in urban, rural and remote, and underserved populations. Addressing the rising congenital syphilis rates in Canada effectively will require a multipronged, holistic, and collaborative approach that promotes equitable living conditions and prenatal care and that supports community-led responses centering the needs and voices of people with lived experience at their core.

## Data Availability

The original contributions presented in the study are included in the article/Supplementary material, further inquiries can be directed to the corresponding author.
