# Supplementary material for: Rising congenital syphilis rates in Canada, 1993–2022
Source: Front Public Health. 2025 Jan 17;12:1522671. doi: 10.3389/fpubh.2024.1522671 (PMC11783095; doi:10.3389/fpubh.2024.1522671)
Supplement: Supplementary file 1 [file Data_Sheet_1.docx]

# Hausse des taux de syphilis congénitale au Canada, 1993 à 2022

## Résumé

**Introduction :** Le nombre de cas de syphilis congénitale précoce confirmée a fortement augmenté au Canada ces dernières années, en particulier depuis 2018, pour atteindre le niveau le plus élevé jamais enregistré depuis le début de la déclaration nationale en 1993. Nous avons analysé les données nationales sur la syphilis congénitale précoce confirmée de 1993 à 2022 pour décrire les tendances épidémiologiques de la syphilis congénitale précoce confirmée au Canada de 1993 à 2022.

**Méthodes :** Les données de 1993 à 2017 proviennent de la surveillance de routine effectuée par l'entremise du Système canadien de surveillance des maladies à déclaration obligatoire, et les données de 2018 à 2022 proviennent de la surveillance rehaussée effectuée par l'entremise d’un groupe de travail fédéral-provincial-territorial. Le nombre de cas et les taux ont été calculés à l'échelle nationale et par province et territoire. Les données sur la syphilis infectieuse de la même période pour les femmes en âge de procréer ont également été analysées.

**Résultats :** Le taux national de syphilis congénitale précoce confirmée était 127 fois plus élevé en 2022 qu'en 1993, passant de 0,3 à 32,7 cas pour 100 000 naissances vivantes. Le nombre de cas a commencé à augmenter rapidement en 2018, le nombre de cas le plus élevé observé à ce jour (n=115) se produisant en 2022. Les taux les plus élevés au pays ces dernières années ont été observés en Saskatchewan, au Manitoba, en Alberta et en Ontario. Les taux de syphilis infectieuse parmi les femmes en âge de procréer ont également augmenté rapidement dans ces provinces. Entre 2018 et 2022, le taux national de syphilis congénitale précoce confirmée a augmenté d'environ sept fois et le taux national de syphilis infectieuse a augmenté d'environ deux fois, y compris une augmentation du taux d'environ trois fois et demie parmi les femmes en âge de procréer.

**Discussion :** Ces chiffres représentent des changements considérables dans le portrait épidémiologique de la syphilis au Canada. L'augmentation de la transmission verticale semble être due non seulement à l'augmentation du taux de syphilis infectieuse parmi les femmes en âge de procréer, mais aussi à de multiples déterminants structurels et sociaux de la santé ayant un impact sur les personnes enceintes.
