# Supplementary material for: Rising congenital syphilis rates in Canada, 1993–2022
Source: Front Public Health. 2025 Jan 17;12:1522671. doi: 10.3389/fpubh.2024.1522671 (PMC11783095; doi:10.3389/fpubh.2024.1522671)
Supplement: Supplementary file 2 [file Table_1.docx]

Table S1. National case definition for confirmed early congenital syphilis (2008-2022)(1).

| **Case classification: Laboratory confirmation of infection** |
| --- |
| - identification of *Treponema pallidum*by dark-field microscopy, fluorescent antibody or equivalent examination of material from nasal discharges, skin lesions, placenta, umbilical cord or autopsy material of a neonate (up to four weeks of age)   or   - reactive serology (non-treponemal and treponemal) from venous blood (not cord blood) in an infant/child with clinical, laboratory or radiographic evidence of congenital syphilis* whose mother is without documented evidence of adequate treatment   or   - detection of *T. pallidum* DNA in an appropriate clinical specimen |

*Includes any evidence of congenital syphilis on physical examination (e.g., hepatosplenomegaly), evidence of congenital syphilis on radiographs of long bones, a reactive CSF (cerebrospinal fluid) VDRL (venereal disease research laboratory) test, an elevated CSF cell count or protein without other cause.

(1) Public Health Agency of Canada. Case Definitions for Communicable Diseases under National Surveillance. Can Commun Dis Rep (2009). 35S2. <https://www.canada.ca/content/dam/phac-aspc/migration/phac-aspc/publicat/ccdr-rmtc/09pdf/35s2-eng.pdf>
