# Supplementary material for: Rising congenital syphilis rates in Canada, 1993–2022
Source: Front Public Health. 2025 Jan 17;12:1522671. doi: 10.3389/fpubh.2024.1522671 (PMC11783095; doi:10.3389/fpubh.2024.1522671)
Supplement: Supplementary file 3 [file Table_2.docx]

Table S2. Number and rates of reported cases of confirmed early congenital syphilis and rates of reported cases of infectious syphilis among females 15 to 39 years old in Canada, 1993-2022.

| **Year** | **Total national cases of confirmed early congenital syphilis** | **Rate of confirmed early congenital syphilis per 100,000 live births** | **Percent (%) change in rate of confirmed early congenital syphilis from previous year** | **Rate of infectious syphilis per 100,000 females 15-39 years old** | **Percent (%) change in rate of infectious syphilis in females 15-39 years old from previous year** |
| --- | --- | --- | --- | --- | --- |
| **1993** | **1** | **0.3** | **NC** | **1.1** | **NC** |
| **1994** | **4** | **1.0** | **303** | **1.0** | **-14** |
| **1995** | **2** | **0.5** | **-49** | **0.7** | **-23** |
| **1996** | **1** | **0.3** | **-48** | **0.7** | **-7** |
| **1997** | **3** | **0.9** | **215** | **0.8** | **11** |
| **1998** | **1** | **0.3** | **-66** | **0.9** | **22** |
| **1999** | **1** | **0.3** | **2** | **1.0** | **4** |
| **2000** | **2** | **0.6** | **106** | **0.8** | **-16** |
| **2001** | **1** | **0.3** | **-51** | **1.4** | **69** |
| **2002** | **4** | **1.2** | **306** | **1.5** | **7** |
| **2003** | **2** | **0.6** | **-51** | **2.2** | **50** |
| **2004** | **1** | **0.3** | **-50** | **1.8** | **-19** |
| **2005** | **9** | **2.6** | **783** | **2.4** | **33** |
| **2006** | **6** | **1.7** | **-36** | **2.4** | **2** |
| **2007** | **7** | **1.9** | **13** | **2.1** | **-12** |
| **2008** | **5** | **1.3** | **-31** | **2.6** | **22** |
| **2009** | **10** | **2.6** | **99** | **2.1** | **-20** |
| **2010** | **5** | **1.3** | **-49** | **2.1** | **2** |
| **2011** | **5** | **1.3** | **0** | **1.6** | **-25** |
| **2012** | **3** | **0.8** | **-41** | **1.4** | **-14** |
| **2013** | **1** | **0.3** | **-66** | **2.1** | **50** |
| **2014** | **1** | **0.3** | **-1** | **2.3** | **10** |
| **2015** | **4** | **1.0** | **302** | **2.6** | **16** |
| **2016** | **4** | **1.0** | **0** | **4.3** | **63** |
| **2017** | **8** | **2.1** | **103** | **6.2** | **45** |
| **2018** | **17** | **4.5** | **114** | **19.5** | **214** |
| **2019** | **53** | **14.2** | **213** | **38.2** | **96** |
| **2020** | **51** | **14.1** | **-1** | **41.0** | **8** |
| **2021** | **109** | **29.4** | **108** | **60.6** | **48** |
| **2022** | **115** | **32.7** | **11** | **67.8** | **12** |

NC: non-computable. Canadian Notifiable Disease Surveillance System (CNDSS) data collection on congenital syphilis commenced in 1993, thus the percent change from the previous year is not calculable.
