# Supplementary material for: Rising congenital syphilis rates in Canada, 1993–2022
Source: Front Public Health. 2025 Jan 17;12:1522671. doi: 10.3389/fpubh.2024.1522671 (PMC11783095; doi:10.3389/fpubh.2024.1522671)
Supplement: Supplementary file 4 [file Table_3.docx]

Table S3. Case counts and rates of infectious syphilis among females 15 to 39 years old, stratified by age group, in Canada, 2018-2022.

|  | **Count** | | | | **Rate** | | | |
| --- | --- | --- | --- | --- | --- | --- | --- | --- |
| **AB** | | | | | | | | |
| **Year** | **15-19 years** | **20-24 years** | **25-29 years** | **30-39 years** | **15-19 years** | **20-24 years** | **25-29 years** | **30-39 years** |
| **2018** | **82** | **129** | **158** | **188** | **67.4** | **97.7** | **99.4** | **55.2** |
| **2019** | **100** | **235** | **259** | **340** | **81.1** | **177.1** | **164.9** | **98.6** |
| **2020** | **101** | **240** | **244** | **332** | **81.0** | **179.3** | **157.1** | **94.8** |
| **2021** | **152** | **284** | **331** | **459** | **122.1** | **216.0** | **220.9** | **131.2** |
| **2022** | **124** | **279** | **305** | **569** | **96.3** | **210.7** | **205.9** | **162.3** |
| **SK** | | | | | | | | |
|  | **15-19 years** | **20-24 years** | **25-29 years** | **30-39 years** | **15-19 years** | **20-24 years** | **25-29 years** | **30-39 years** |
| **2018** | **DNS** | **10** | **DNS** | **DNS** | **DNS** | **28.4** | **DNS** | **DNS** |
| **2019** | **21** | **55** | **33** | **51** | **62.1** | **156.8** | **84.4** | **62.7** |
| **2020** | **71** | **138** | **110** | **132** | **210.9** | **395.2** | **288.1** | **160.2** |
| **2021** | **128** | **242** | **239** | **298** | **382.8** | **702.3** | **649.4** | **363.4** |
| **2022** | **91** | **261** | **295** | **399** | **266.1** | **756.5** | **817.5** | **486.8** |
| **MB** | | | | | | | | |
|  | **15-19 years** | **20-24 years** | **25-29 years** | **30-39 years** | **15-19 years** | **20-24 years** | **25-29 years** | **30-39 years** |
| **2018** | **53** | **105** | **89** | **94** | **127.0** | **234.5** | **190.2** | **103.7** |
| **2019** | **103** | **250** | **267** | **272** | **246.9** | **550.8** | **564.6** | **295.4** |
| **2020** | **97** | **187** | **210** | **258** | **236.1** | **409.2** | **443.1** | **276.2** |
| **2021** | **101** | **220** | **245** | **329** | **253.3** | **473.3** | **521.7** | **351.3** |
| **2022** | **126** | **224** | **255** | **305** | **308.6** | **468.9** | **538.1** | **323.3** |
| **ON** | | | | | | | | |
|  | **15-19 years** | **20-24 years** | **25-29 years** | **30-39 years** | **15-19 years** | **20-24 years** | **25-29 years** | **30-39 years** |
| **2018** | **10** | **23** | **18** | **24** | **2.4** | **4.8** | **3.7** | **2.5** |
| **2019** | **DNS** | **37** | **40** | **42** | **DNS** | **7.6** | **7.9** | **4.3** |
| **2020** | **DNS** | **35** | **60** | **85** | **DNS** | **7.1** | **11.5** | **8.4** |
| **2021** | **19** | **66** | **94** | **169** | **4.8** | **13.9** | **18.0** | **16.6** |
| **2022** | **21** | **83** | **117** | **214** | **5.1** | **17.2** | **21.9** | **20.4** |

Note: data are based on the four provinces that have reported 10 or more cases of confirmed early congenital syphilis in Canada: AB, SK, MB, ON. Provinces and territories reporting fewer than 10 cases of confirmed early congenital syphilis were excluded from the Table.
DNS: data not shown to reduce the risk of identifying individuals (low case counts). AB, Alberta; SK, Saskatchewan; MB, Manitoba; ON, Ontario.
