# Supplementary material for: Rising congenital syphilis rates in Canada, 1993–2022
Source: Front Public Health. 2025 Jan 17;12:1522671. doi: 10.3389/fpubh.2024.1522671 (PMC11783095; doi:10.3389/fpubh.2024.1522671)
Supplement: Supplementary file 5 [file Table_4.docx]

Table S4. Rates of reported cases of infectious syphilis by sex and male-to-female rate ratio in Canada, 2022.

| **Province or territory** | **Total rate per 100,000 population** | **Rate per 100,000 males** | **Rate per 100,000 females** | **Male-to-female rate ratio** |
| --- | --- | --- | --- | --- |
| **British Columbia** | **27.8** | **42.7** | **12.8** | **3.3** |
| **Alberta** | **74.2** | **79.0** | **69.0** | **1.1** |
| **Saskatchewan** | **188.9** | **174.0** | **204.1** | **0.9** |
| **Manitoba** | **136.1** | **121.5** | **150.5** | **0.8** |
| **Ontario** | **23.7** | **39.7** | **7.6** | **5.2** |
| **Québec** | **14.6** | **24.4** | **4.6** | **5.3** |
| **New Brunswick** | **4.7** | **DNS** | **DNS** | **4.5*** |
| **Nova Scotia** | **4.5** | **DNS** | **DNS** | **14.7*** |
| **Prince Edward Island** | **DNS** | **DNS** | **DNS** | **1.6*** |
| **Newfoundland and Labrador** | **2.6** | **DNS** | **DNS** | **6.1*** |
| **Yukon** | **140.4** | **160.7** | **120.0** | **1.3** |
| **Northwest Territories** | **370.3** | **374.5** | **365.9** | **1.0** |
| **Nunavut** | **113.6** | **122.0** | **105.0** | **1.2** |
| **Canada** | **36.65** | **47.0** | **25.9** | **1.8** |

Note: total case counts and rates presented nationally include reported cases among all sexes.
DNS: data not shown to reduce the risk of identifying individuals (low case counts).
*Interpret with caution as this ratio is based on small numbers.
